# Comparative Molecular Modelling of Capsular Polysaccharide Conformations in *Streptococcus suis* Serotypes 1, 2, 1/2 and 14 Identifies Common Epitopes for Antibody Binding

**DOI:** 10.3389/fmolb.2022.830854

**Published:** 2022-02-08

**Authors:** Michelle M. Kuttel

**Affiliations:** Department of Computer Science, University of Cape Town, Cape Town, South Africa

**Keywords:** antigen, capsular polysaccharide, carbohydrate epitopes, conformation, molecular modelling and simulation, *Streptococcus suis*, cross protection

## Abstract

*Streptococcus suis* is an encapsulated, commensal, potentially pathogenic bacterium that infects swine globally and causes sporadic life-threatening zoonotic septicemia and meningitis infections in humans. The capsular polysaccharide is a primary virulence factor for *S. suis.* As *S. suis* serotype 2 is the most prevalent serotype globally, the serotype 2 CPS is the primary target of current efforts to develop an effective glycoconjugate veterinary vaccine against *S. suis.* Possible cross-protection with related serotypes would broaden the coverage of a vaccine. The CPS in serotypes 2 and 1/2 differ at a single residue (Gal versus GalNAc), and both are similar to serotypes 1 and 14: all contain a terminal sialic acid on a side chain. However, despite this similarity, there is complex pattern of cross-protection for these serotypes, with varying estimations of the importance of sialic acid in a protective epitope. Further, a pentasaccharide without the terminal sialic acid has been identified as minimal epitope for serotype 2. Here we use molecular simulation to model the molecule conformations of the CPS in serotypes 2, 1/2, 1 and 14, as well as three vaccine candidate oligosaccharides. The common epitopes we identify assist in rationalizing the apparently contradictory immunological data and provide a basis for rational design of *S. suis* vaccines in the future.

## Introduction

*Streptococcus suis* is an encapsulated, commensal, potentially pathogenic bacterium that primarily inhabits the upper respiratory tract of pigs. *S. suis* is not only a principal cause of death in piglets (Gottschalk 2011), with consequent economic losses to the swine industry, but is also responsible for sporadic zoonotic meningitis, septicemia and other infections in humans who have been in contact with pigs, or consumed raw pork products (Goyette-Desjardins et al., 2014). Recent deadly outbreaks of infections in Asia mean that *S. suis* is now considered to be a serious emerging zoonotic agent (Dutkiewicz et al., 2018). Therefore, development of effective treatments against *S. suis* is increasingly necessary, most particularly a veterinary vaccine to limit disease in swine and consequently transmission to humans (Segura 2015; Segura et al., 2016). Vaccination reduces the consumption of antibiotics and hence the development of antimicrobial resistance.

The capsular polysaccharide (CPS) is a primary virulence factor for *S. suis* that protects the bacteria from host phagocytosis (Gottschalk et al., 2010; Auger et al., 2018), but is also a target for anti-body mediated host defense and thus a key component of many modern anti-bacterial vaccines. The poor immunogenicity of polysaccharide vaccines can be overcome by immunization with a glycoconjugate, which comprises carbohydrate antigens conjugated to a suitable protein carrier (Astronomo and Burton 2010). Glycoconjugate vaccines based on capsular polysaccharides have been highly successful against infection by *Haemophilus influenzae*, *Streptococcus pneumoniae* and *Neisseria meningitidis* serotypes in humans (Lockhart et al., 2018) and are a promising avenue for development of a veterinary vaccine against *S. suis* (Goyette-Desjardins et al., 2016; Goyette-Desjardins et al., 2019).

*S. suis* strains are classified into serotypes on the basis of the CPS antigenicity. The *S. suis* CPS is diverse: based on CPS antigens, 35 serotypes of *S. suis* have been identified and healthy pigs carry multiple *S. suis* serotypes in their upper respiratory tract (Dutkiewicz et al., 2017). However, most porcine infections are caused by a small subset of serotypes: in order of decreasing prevalence, serotypes 2, 9, 3, 1/2, 8, 7, 4, 22, 5, and 1 (Goyette-Desjardins et al., 2014). In humans, *S. suis* serotype 2 is the most virulent strain worldwide, followed by serotype 14 (Goyette-Desjardins et al., 2014; Segura et al., 2016). The focus of *S. suis* conjugate vaccine development has therefore been thus primarily on serotype 2 (Goyette-Desjardins et al., 2016; Goyette-Desjardins et al., 2019).

Since 2010, CPS repeating unit (RU) structures have been determined for *S. suis* serotypes 1, 1/2, 2, 7, 8, 9, 14 and 18 (Goyette-Desjardins et al., 2020). The focus of this work is a comparison of the CPS from four closely related *S. suis* serotypes: 1, 1/2, 2 and 14 (Van Calsteren et al., 2010; Van Calsteren et al., 2013; Van Calsteren et al., 2016). These serotypes have very similar CPS compositions (henceforth labeled Ss2, Ss1/2, Ss1 and Ss14, respectively) which are as follows (for comparison purposes, differences from Ss14 are highlighted in **bold**).

Ss2: **→4**)[αdNeu5Ac(2→6)βdGal(1→4)βdGlcNAc(1→3)]βdGal(1→**4**)**[α** **d** **Gal**(**1→3)]β** **l** **Rha** (1→4)βdGlc(1→

Ss1/2: **→4**)[αdNeu5Ac(2→6)βdGal**NAc**(1→4)βdGlcNAc(1→3)]βdGal(1→**4)[α** **d** **Gal**(**1→3)]β** **l** **Rha** (1→4)βdGlc(1→

Ss1: →6)[αdNeu5Ac(2→6)βdGal**NAc**(1→4)βdGlcNAc(1→3)]βdGal(1→3)βdGal(1→4)βdGlc(1→

Ss14: →6)[αdNeu5Ac(2→6)βdGal(1→4)βdGlcNAc(1→3)]βdGal(1→3)βdGal(1→4)βdGlc(1→

**FIGURE 1:**
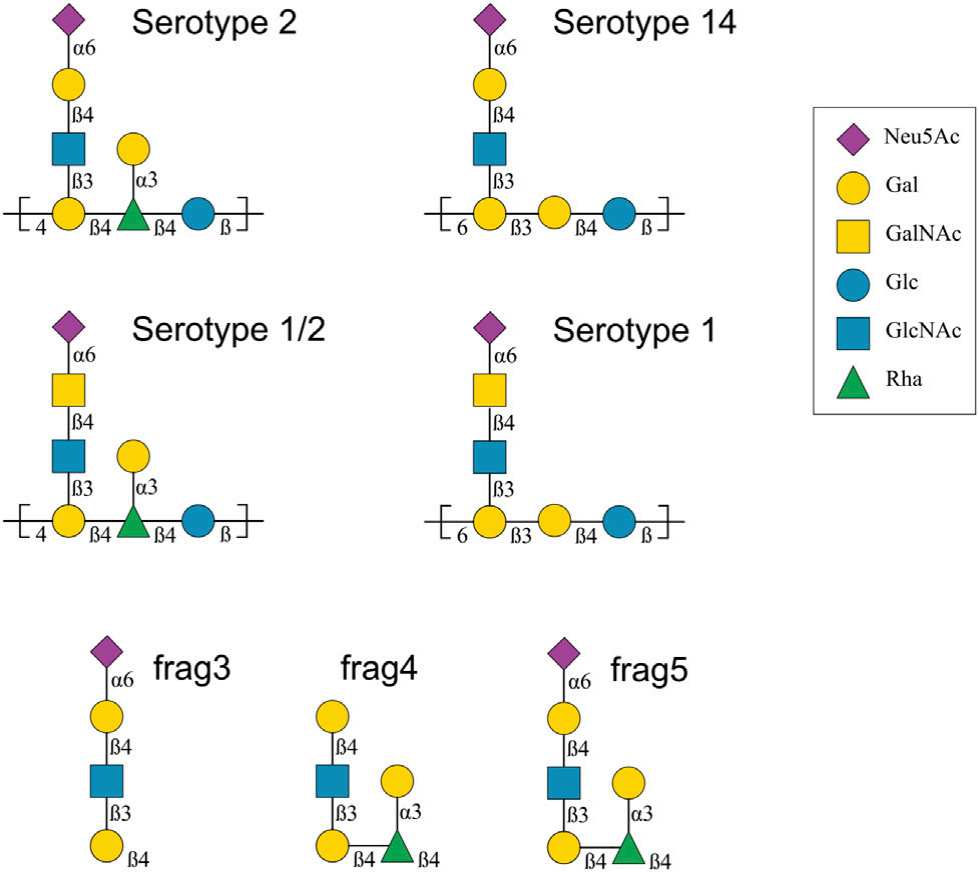
Capsular polysaccharide repeating unit structures for *Streptococcus suis* serotypes 2, 14, 1/2 and 1 and 14 (Van Calsteren et al., 2010; Van Calsteren et al., 2013; Van Calsteren et al., 2016), as well as the structure of three Ss2 oligosaccharide vaccine candidates, labelled frag3, frag4 and frag5 (Zhang et al., 2021). The graphical representations use the Symbol Nomenclature for Glycans (SNFG) (Varki et al., 2015). Abbreviations: d-glucose (Glc), d-galactose (Gal), N-acetyl-d-glucosamine (GlcNAc), N-acetyl-d-galactosamine (GalNAc), l-rhamnose (Rha), N-acetyl-d-neuraminic acid (Neu5Ac).

These CPS structures are also shown with symbols in Figure 1. Ss2 and Ss1/2 have a seven-residue RU, whereas Ss14 and Ss1 have a six-residue RU. The backbone compositions are the same in Ss1 and Ss14, →6)βdGal(1→3)βdGal(1→4)βdGlc(1→, and in Ss2 and Ss1/2, →4)βdGal(1→4)βlRha(1→4)βdGlc(1→. All four CPS have a three-residue side chain comprising a terminal sialic acid (Neu5Ac) linked to O-6 of either α-d-galactose (Ss2 and Ss14) or α-d-N-acetylgalactosamine (Ss1/2 and Ss1). Ss2 and Ss1/2 also have an additional α-d-galactose side chain.

To design an effective conjugate vaccine, it is important to identify antigenic epitopes that generate the production of antibodies able to protect the host from a pathogen (Anish et al., 2014). In particular, identification of protective carbohydrate epitopes has been recognized as a crucial step in the development of a glycoconjugate vaccine against *S. suis* (Goyette-Desjardins et al., 2019). However, this is not a straightforward task, as direct experimental determination of the molecular conformation of carbohydrates is often confounded by the flexibility of this molecular class (Woods 2018). Further, immunogenicity studies of *S. suis* have shown complex cross-reactivity patterns, with varying estimations of the importance of sialic acid in a protective epitope, as follows. Serological analyses of cross-reaction between Ss2, Ss1/2, Ss1 and Ss14 using purified CPS and rabbit type-specific sera showed cross-reaction of Ss1/2 with Ss1 and Ss2, as well as between Ss1 and Ss14, and suggested that the side chain is an important epitope for Ss2, Ss1/2 and Ss1, but not for Ss14 (Van Calsteren et al., 2016). Further, while the terminal sialic acid appeared to be immunodominant for Ss2 and Ss1/2, it seemed to play a limited role in Ss1. A subsequent, recent study of four murine monoclonal antibodies (mAbs) raised against an Ss2 glycoconjugate provided evidence of a range of *S. Suis* CPS epitopes, only some of which elicit protective antibodies: one mAb (9E7) cross-reacted with all of Ss2, Ss1/2, Ss1 and Ss14; another (Z3) with Ss2, Ss1/2 and Ss1; the third (13C8) with just Ss2 and Ss14 (an unexpected result based on the published serological work); and the fourth (16h11) was Ss2 specific, with no cross-reactivity (Goyette-Desjardins et al., 2019). However, mAb 16H11 was not found to be protective. In addition, all four mAbs showed strong specificity toward the sialylated side chain chains of the CPSs, but not against their desialylated counterparts, indicating that the terminal sialic acids forms part of the protective epitope for these mAb. However, prior work demonstrated that the sialic acid moiety is not essential for CPS recognition by an Ss2-specific antibody (Lecours et al., 2012). To further complicate matters, recent investigation of three oligosaccharide lead antigens for Ss2 (labelled frag3, frag4 and frag5 in Figure 1) showed efficacy for a penta- and a hexasaccharide (frag4 and frag5), but not the tetrasaccharide (frag3), indicating that sialic acid is not a necessary component of a minimal protective epitope for Ss2 (Zhang et al., 2021).

As there are currently no experimental data available on the molecular conformations of this group of CPS antigens, molecular modelling can provide useful information on which epitopes are exposed and thus likely to be recognized by antibodies (Kuttel and Ravenscroft 2018). We have performed a range of comparative modelling investigations of microbial CPS to explain clinical findings, most recently of *Haemophilus influenzae* (Richardson et al., 2021) and *Cryptococcus neoformans* (Kuttel et al., 2020). Here we undertake a comparative modelling study of the Ss2, Ss1/2, Ss1 and Ss14 CPS as well as the three synthetic vaccine candidate oligosaccharides, comparing the predicted molecular flexibility and conformations to identify and contrast the probable protective epitopes for these four *S. suis* CPS. We aim with this work to explain the conflicting immunological data and provide a basis for rational design of *S. suis* vaccines in the future.

## Materials and Methods

We ran molecular dynamics (MD) simulations in aqueous solution for CPS chains of 6 RU for each of Ss2, Ss1/2, Ss1 and Ss14, as well as for the three oligosaccharide lead antigens for Ss2 (labelled frag3, frag4 and frag5 in Figure 1) that have been synthesized and tested (Zhang et al., 2021). Note that the aminopentyl spacer at the reducing end of the synthesized antigen used for creating microarrays and protein conjugates was not included in the modeling. The trajectories produced by these seven simulations were then analyzed and compared, as detailed below.

### Glycosidic Linkage Conformations

In a carbohydrate, the orientation of a two-bond glycosidic linkage is conveniently described by the values of two torsion angles, φ and ψ. In this work these are defined as φ = H1-C1-O1-C_x_’ and ψ = C1-O1-C_x_’-H_x_’, where X ∈ (3, 4) denotes the linkage position. These definitions are analogous to φ_H_ and ψ_H_ in IUPAC nomenclature; conveniently locate the global minimum for most glycosidic linkages near *φ*, ψ = 0, 0; and are consistent with our previous work. For the αdNeu5Ac(2→6)βdGal three-bond linkage, three torsions are necessary and are defined as φ = C1-C2-O6′-C6′, ψ = C2-O6′-C6′-C5′ and ω = O6′-C6′-C5′-O5’.

### MD Simulation Protocol

All simulations were run with the NAMD software package (Phillips et al., 2005), employing CUDA extensions to enable calculation of long-range electrostatic potentials and non-bonded forces on graphics processing units (Stone et al., 2007).

Initial structures of 6 RU for each of Ss2, Ss1/2, Ss1 and Ss14, as well three oligosaccharide lead antigens frag3, frag4 and frag5 (Figure 1), were built with our in-house CarbBuilder software (Kuttel et al., 2016) version 1.2.40 which employs the psfgen tool to create “protein structure” (psf) files for simulation with CHARMM force fields. The saccharides were modeled with the CHARMM36 additive force field for carbohydrates (Guvench et al., 2011). The starting conformations produced by CarbBuilder were each subjected to 10,000 steps of standard NAMD minimization in vacuum and then placed into a cubic water box with the *solvate* command from the Visual Molecular Dynamics (VMD) software (Humphrey et al., 1996). The cubic water boxes for the 6 RU structures had side lengths of 80 Å, those for the smaller antigens had side lengths of 40 Å. The TIP3P model (Jorgensen et al., 1983) was used to simulate water. Randomly distributed sodium counter ions were added to the charged systems to neutralize the negative charge on the sialic acid residues: one Na+ was added to the frag3 and frag5 systems and six to Ss1, Ss1/2, Ss1 and Ss14. The frag4 system is missing the sialic residue and therefore did not require a counter ion.

Each system was then gradually heated through a protocol of 5 K incremental temperature reassignments from 10 to 300 K, with 500 steps of NAMD minimization and 8,000 steps of MD after each temperature reassignment. MD simulations were then run for 1 μs for both Ss2 and Ss1/2, and 1.4 μs for the more flexible Ss1 and Ss14. The smaller frag3, frag4 and frag5 oligosaccharides were each simulated for 0.5 μs.

In each simulation, equations of motion were integrated using a Leap-Frog Verlet integrator with a step size of 1 fs and periodic boundary conditions. Simulations were performed under isothermal-isobaric (nPT) conditions at 300 K maintained using a Langevin piston barostat (Feller et al., 1995) and a Nose-Hoover (Nosé and Klein 1983; Hoover 1985) thermostat. Long-range electrostatic interactions were treated using particle mesh Ewald (PME) summation, with k = 0.20 Å^-1^ and a 1 Å PME grid spacing. Non-bonded interactions were truncated with a switching function applied between 12.0 and 15.0 Å to groups with integer charge. The 1–4 interactions were not scaled, in accordance with the CHARMM force field recommendations.

For all simulations, structures were collected at intervals of 250 fs for analysis.

### Simulation Convergence

Convergence of the CPS simulations was measured with the block standard error method (Grossfield and Zuckerman 2009) applied to the end-to-end distance (Supplementary Figures S1, S2). Block standard averaging was implemented with in-house Python scripts.

For all simulations, the blocked standard error estimates of the correlation times for end-to-end distance indicated convergence of the simulations: approximately 10 ns for Ss2 and Ss1/2; 30 ns for Ss1 and Ss14. The estimated number of independent samples also were much greater than one: 109 (Ss2); 81 (Ss1/2); 51 (Ss14); and 49 (Ss1). Note that our designated equilibration time of 200 ns is in all cases considerably greater than the correlation time.

### Data Analysis

Analysis of the simulations used time series frames 25 ps apart. Molecular conformations extracted from the MD simulations were depicted with VMD, where necessary using the PaperChain visualisation algorithm for carbohydrates to highlight the hexose rings (Cross et al., 2009). Molecular metrics (such as dihedral angles and end-to-end distances) were extracted from the simulation trajectories using VMD’s Tcl scripting interface. Data analyses were performed using in-house Python scripts and plots generated using the Matplotlib graphing library for Python (Hunter 2007). The dihedral angles for each glycosidic linkage were plotted for the dihedrals from RU 3 and RU 4.

Conformations from all MD simulation trajectories were clustered using VMD’s internal *measure cluster* command. Clustering analysis used time series frames 250 ps apart, discarding the first 200 ns as equilibration. For the backbone clustering analysis of the CPS, simulation conformations were first aligned on the ring atoms of the backbone residues in RU 3–4 (the middle two repeats in the 6 RU chain, excluding the side chains). Then all conformations were clustered into families according to a root mean square deviation (rmsd) fit (cutoff of 5.5 Å) to the saccharide ring atoms of the backbone residues in RU 2–5 (excluding the more flexible first and last RU, and the side chains). For clustering analysis of the side chains, simulation conformations were first aligned on the ring atoms of the βdGal branch point residue in RU 4. Then all conformations were clustered into families according to a rmsd fit (cutoff of 2.0 Å) to the ring atoms of all residues in RU 4, excluding the βdGlc backbone residue. Similarly, the antigens (frag3, frag4 and frag5) were clustered by first aligning the conformations on the ring atoms of βdGlcNAc and the βdGal residues (excluding αdNeu5Ac and αlRha). Then all conformations were clustered into families according to a rmsd fit (cutoff of 2.0 Å) to the ring atoms of all residues in the fragment.

The solvent accessible surface area of each molecule was calculated with VMD’s built in “measure sasa” command with a probe radius of 1.4 Å. The ratio of the exposed backbone residues to the entire exposed molecule surface was then calculated to estimate the surface area available for potential antibody binding.

## Results

Our analysis of the MD simulations starts with a broad comparison of the CPS backbone across the *S. suis* serogroups Ss2, Ss1/2, Ss14 and Ss1: we measure the molecular extension and flexibility and characterize the dominant backbone conformations. We then focus on a characterization of the side chain conformations in each CPS, which are compared to the conformations of the Ss2 antigens frag3, frag4 and frag5.

### CPS Backbone

**FIGURE 2:**
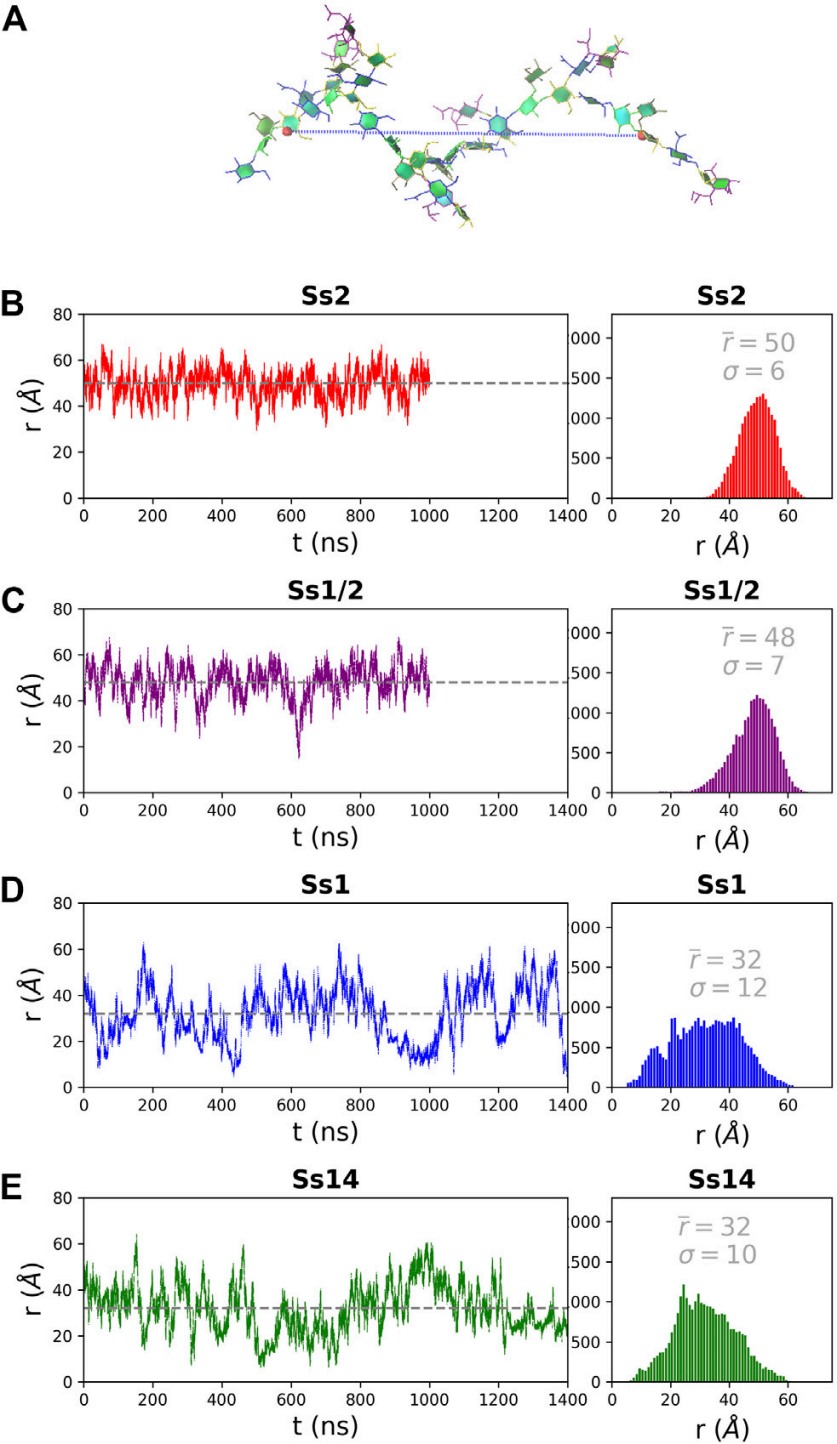
The end-to-end distance, *r*, for the 6 RU *S. suis* CPS is here defined as the distance (Å) from the ring O5 in the backbone βDGal in the first and last RU, labelled in **(A)** on a sample Ss2 conformation. The simulation time series (left column) and corresponding histograms (right column) are graphed for **(B)** Ss2, **(C)** Ss1/2, **(D)** Ss1 and **(E)** Ss14. The first 200 ns of simulation are considered equilibration and are not shown. The average *r* value $\bar{r}$ (plotted as a dashed gray line on the time series graphs) and the standard deviation σ are listed on each histogram.

The structure of the CPS backbone differs: Ss2 and Ss1/2 have a backbone containing only β(1→4) linkages, while Ss14 and Ss1 have a backbone with a flexible β(1→6) linkage (Figure 1). Simulation is a useful tool for investigating the effects of this change on the flexibility and conformation of the CPS. The fluctuation in molecular end-to-end distance, *r*, over the course of a simulation is a commonly used measure of chain extension and flexibility in carbohydrates. Here we define *r* for all 6 RU CPS chains as the distance between the ring O5 atoms in the backbone β**-** d**-** Gal in the first and last RU, shown for Ss2 in Figure 2A. The *r* time series plots (Figure 2, left column) and corresponding histograms (Figure 2, right column) for Ss2, Ss1/2, Ss14 and Ss1 reveal significant differences between the extension and flexibility of the →4)βdGal(1→4)βlRha(1→4)βdGlc(1→ backbone (Ss2 and Ss1/2) and the →6)βdGal(1→3)βdGal(1→4)βdGlc(1→ backbone (Ss14 and Ss1). Comparison of the average, $\bar{r}$ , and the variance, *σ*, of *r* across the CPS show that Ss2 ( $\bar{r}$ = 50 Å, *σ* = 5, Figure 2B) and Ss1/2 ( $\bar{r}$ = 48 Å, *σ* = 7, Figure 2C) are more extended and much less flexible than Ss1 ( $\bar{r}$ = 32 Å, *σ* = 12, Figures 2D,Ss14 ( $\bar{r}$ = 32 Å, *σ* = 10, Figure 2E). The roughly Gaussian distribution of *r* in Ss2 and, to a lesser extent, Ss1/2, indicates a well-defined, albeit flexible, backbone conformation. In contrast, the plot of *r* for Ss1 and Ss14 is evidence of dynamic conformational changes that are more in keeping with random coil behavior. This is to be expected, as Ss14 and Ss1 have a three bond glycosidic linkage βdGlc(1→6)βdGal, which is considerably more flexible than the βdGlc(1→4)βdGal linkage in Ss2 and Ss1/2. However, comparison of the distribution of the backbone glycosidic linkage conformations across these two groups (see Supplementary Material, Supplementary Figures S3, S4) reveals that the source of flexibility is not only the (1→6) linkages: all the backbone glycosidic linkages are very flexible in Ss14 and Ss1 (Supplementary Figure S4) as compared to the much more constrained linkages in Ss2 and Ss1 (Supplementary Figure S3). Further, for Ss2 and Ss1/2, the αdGal side chain is in close proximity to the βdGal branch point; intermolecular interactions between these two residues restrict the rotation of the βdGal(1→4)βlRha backbone glycosidic linkage. Therefore, all the residue and linkages changes in the Ss1/Ss14 backbone relative to the Ss2/Ss1/2 backbone increase the flexibility of the CPS chain. This increased chain flexibility of Ss1 and Ss14 is expected to correspond to a less viscous CPS solution than for Ss2 and Ss1/2.

**FIGURE 3:**
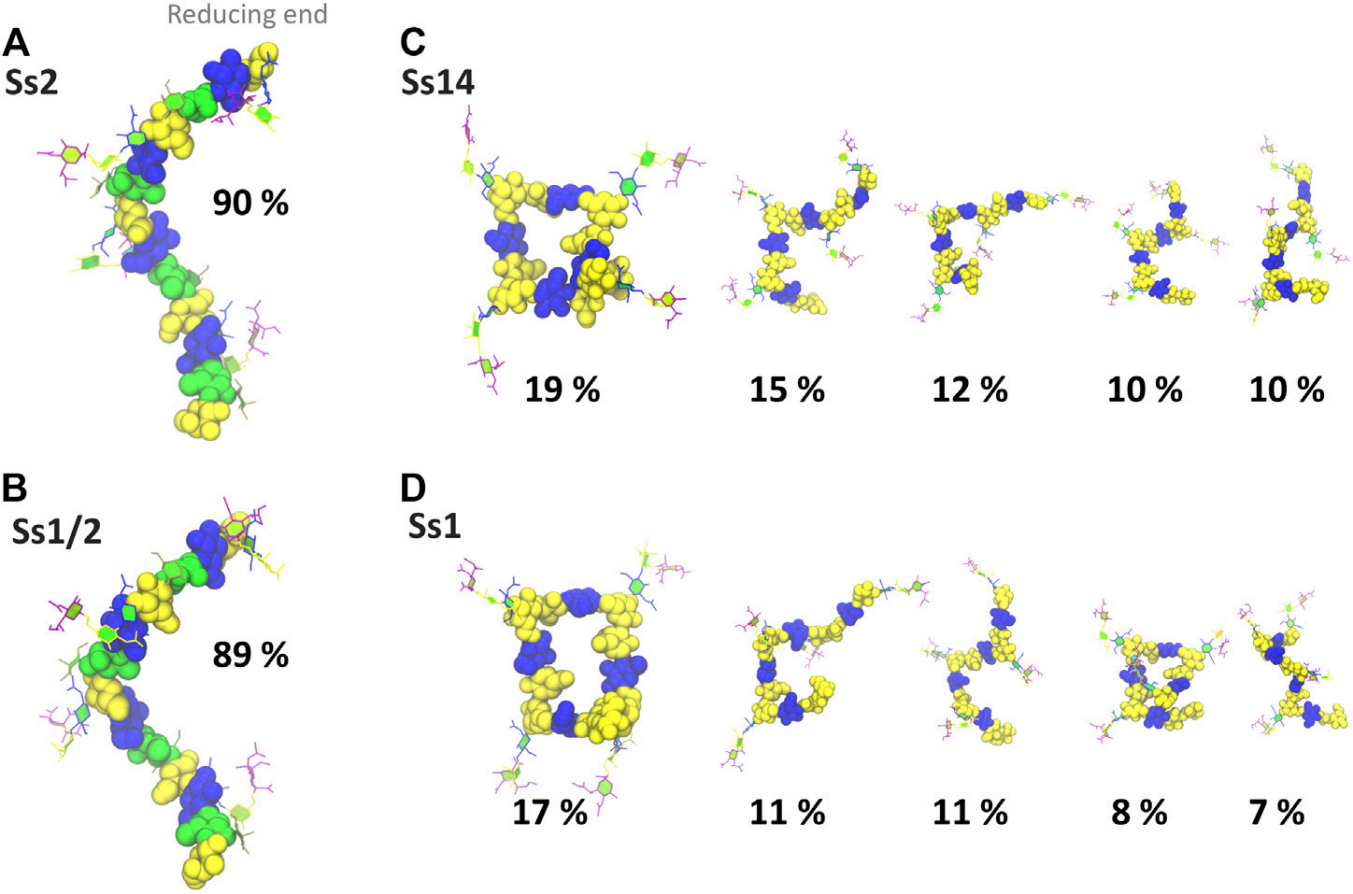
Main conformational families and associated percentages of the CPS molecules shown with the side chains in the PaperChain visualisation and the CPS backbone in a space-filling representation. Representative conformations for RU 2–5 (excluding the first and last RU) are shown for **(A)** Ss2; **(B)** Ss1/2; **(C)** Ss14; and **(D)** Ss1. Conformations are scaled according to their relative percentage occupancies, and clusters less than 6% are not shown. The residues in all representations are coloured in keeping with the SNFG, as follows: βdGal and βdGalNAc—yellow; αdGal—brown; βdGlc—blue; βlRha—green; αdNeu5Ac—purple.

In addition to a significant decrease in flexibility, Ss2 and Ss1/2 have markedly different backbone conformations to Ss14 and Ss1, as shown in Figure 3. In both Ss2 (Figure 3A) and Ss1/2 (Figure 3B), the dominant backbone conformation comprises ∼90% of the simulation and is a flexible, extended helix, with 9–12 backbone residues per turn (3-4 RU). In this helical conformation, both side chains are exposed on the outside of the helix and are therefore potential epitopes for antibody binding. In contrast, the backbones in both Ss14 (Figure 3C) and Ss1 (Figure 3D) do not have a single, well-defined conformation, but rather a family of conformations (Figures 3C,D). Interestingly, although the backbone conformations vary, the dominant conformations are similar in Ss14 and Ss1, with the main conformation being a “square” with bends at each of the βdGlc(1→6)βdGal linkages, which is also the point of side chain branching. This has the effect of exposing the side chains, making them clear epitopes for antibody binding. The minor conformations of Ss14 and Ss1 differ, but all feature bends at the βdGlc(1→6)βdGal linkage. These bends also render the backbone epitopes in Ss1 and Ss14 more exposed than in Ss2 and Ss1/2, where the backbone is relatively hidden by the helical structure as well as the additional αdGal(1→3)βlRha side chain. Analysis of the solvent accessible surface area of the CPS shows that the average solvent exposure of the backbone residues relative to molecule as a whole increases from around 30% in the helical Ss2 and Ss1/2, to approximately 40% in Ss1 and Ss14 (see Supplementary Material, Supplementary Figure S5). However, in all the CPS, the sialic acid side chain is highly solvent exposed and therefore the most likely source of epitopes for antibody binding.

### Trisaccharide Side Chain

**FIGURE 4:**
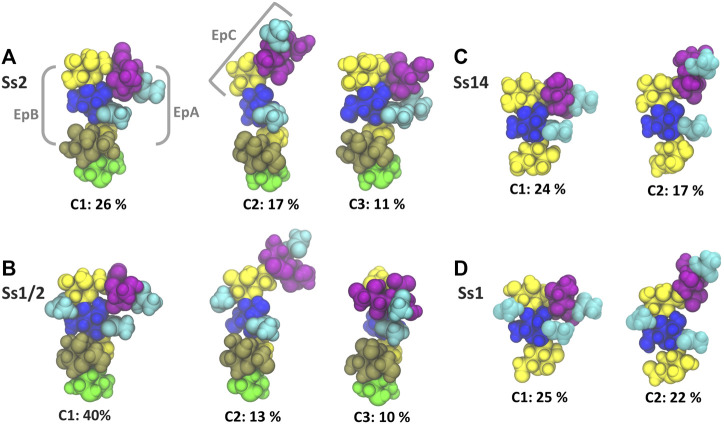
Main conformational families and associated percentages of the side chains in RU 4 for **(A)** Ss2; **(B)** Ss1/2; **(C)** Ss14; and **(D)** Ss1. Conformational clusters of less than 10% occupancy are not shown. Potential epitopes are labelled on Ss2 in grey. The residues in all representations are coloured in keeping with the SNFG, as follows: βdGal and βdGalNAc—yellow; αdGal—brown; βdGlc and βdGlcNAc—blue; βlRha—green; αdNeu5Ac—purple; NAc—cyan.

The dominant conformational cluster (C1) of the trisaccharide side chain is very similar across all four CPS (Figures 4A–D). This conformation features a hairpin bend, with the sialic acid bent towards the CPS backbone and the βdGal/βdGalNAc residue exposed at the end of the side chain. The C1 conformation also brings the N-acetyl groups on αdNeu5Ac and βdGlc into close proximity (colored cyan in Figure 4), forming a common epitope across the four CPS (labelled “EpA” on conformation C1 in Figure 4A). However, this hairpin bend conformation of the side chain also provides a rationale for why sialic acid many not be a necessary component of a minimal protective epitope for Ss2 (Lecours et al., 2012): it is possible for antibody binding may occur on the other side of the side chain. This epitope, labelled “EpB” in Figure 4A, excludes the sialic acid.

The secondary conformational cluster, C2, is also remarkably similar across the CPS: the sialic acid is extended at an angle at the end of the side chain. This conformation creates a new potential conformational epitope for antibody binding, labelled “EpC” in Figure 4A, combining the sialic acid with the adjacent βdGlc residue. However, while the substitution of βdGal in Ss2 and Ss14 with βdGalNAc in Ss1/2 and Ss1 does not affect the dominant conformation of the side chain or the EpA epitope (compare C1 conformations in Figures 4A,B), it does alter the EpB and EpC epitopes considerably. In Ss1 and Ss1/2, the N-acetyl group on C2 of βdGal is highly sovlent exposed on the side chain, increasing the extent of the binding surface to form larger “EpB*” and “EpC*” epitopes.

The flexibility of the trisaccharide side chains differ across the CPS: Ss1/2 has by far the most conformationally defined side chain (with the main C1 conformation at 40%), whereas Ss2, Ss1 and Ss14 have similar C1 frequencies: 26, 25 and 24% C1, respectively. Further, analysis of the glycosidic linkage rotations (Supplementary Material, Supplementary Figure S6) shows that the flexibility of the side chain increases in the order Ss1/2, Ss2, Ss1 and Ss14. In particular, rotation of the βdGlcNAc(1→3)βdGal and, to a lesser extent, the βdGalNAc(1→4)βdGlcNAc linkage is restricted in Ss2 and Ss1/2 as a result of the adjacent constraining αdGal side chain in these CPS.

### Vaccine Candidates

**FIGURE 5:**
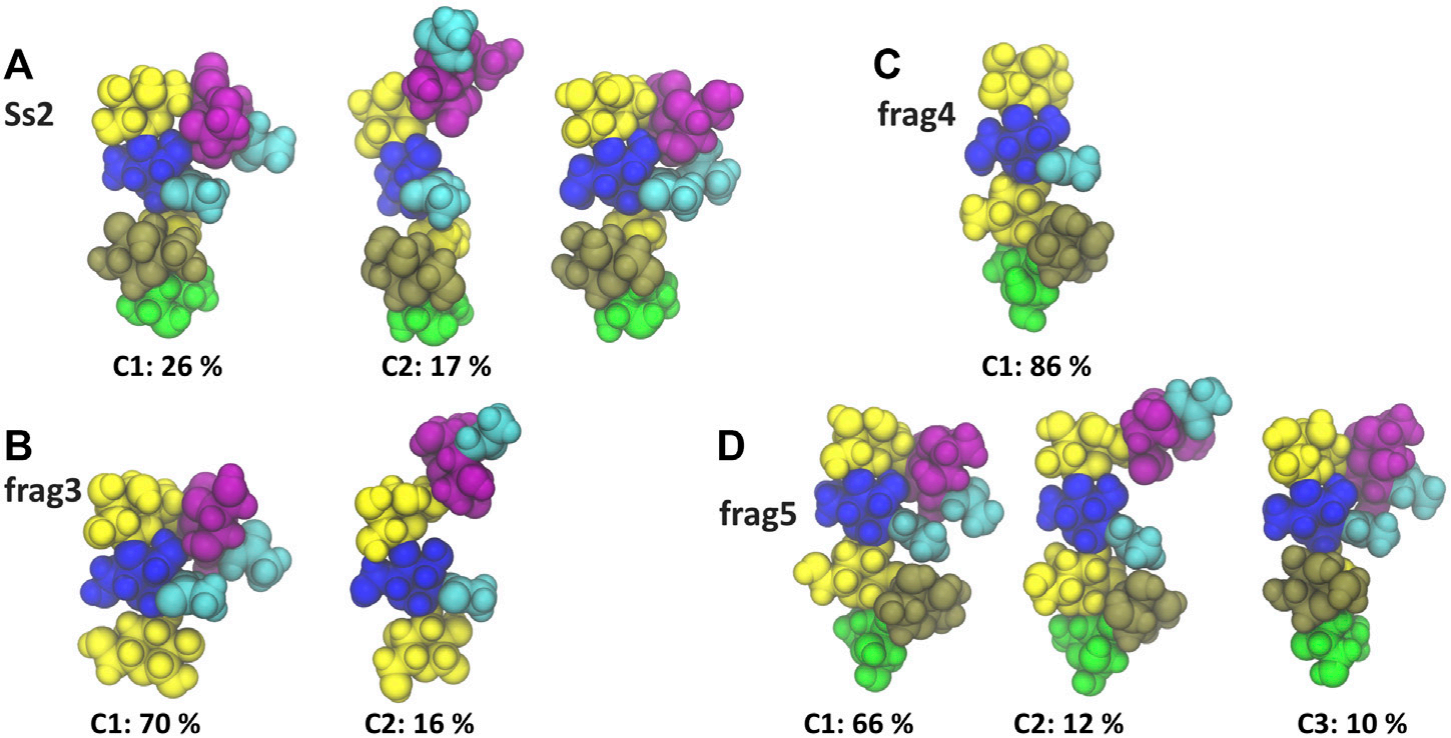
Main conformational families and associated percentages of the sialic side chain in **(A)** RU 4 of Ss2 compared with the dominant conformation of the three Ss2 oligosaccharide vaccine candidates **(B)** frag3, **(C)** frag4 and **(D)** frag5 (Zhang et al., 2021). Conformational clusters with less than 10% occupancy are not shown. The residues in all representations are coloured in keeping with the SNFG, as follows: βdGal and βdGalNAc—yellow; αdGal—brown; βdGlc and βdGlcNAc—blue; βlRha—green; αdNeu5Ac—purple; NAc—cyan.

A comparison of the Ss2 side chain conformations (Figure 5A) with the frag3 tetrasaccharide (Figure 5B), frag4 pentasaccharide (Figure 5C) and frag5 hexasaccharide (Figure 5D) shows that the dominant C1 conformation of the sialic acid side chain is preserved in all the three vaccine candidates (Zhang et al., 2021). All three have similar torsion angle distributions to Ss2 (see Supplementary Material, Supplementary Figure S6). Further, all three preserve the EpB epitope. However, while frag3 and frag5 expose the EpA epitope in the dominant C1 conformation, this epitope is very much altered in frag4, which is missing the sialic acid. The fact that Zhang et al. reported frag4 and frag5 to have similar binding strengths to rabbit antibodies (Zhang et al., 2021) suggests that the EpA epitope is not essential and that the EpB epitope is immunodominant in rabbits. However, it is surprising that frag3 was found to be a poor vaccine candidate in the same study, as this is not explained by our simulation conformations, where frag3 has both the EpA and EpB epitopes (Figure 5B). This suggests that the conformation of the frag3 molecule may in this case be influenced by the aminopentyl spacer which is at the reducing end of the synthesized molecule, but is not modelled here (as it is not currently supported by the CHARMM carbohydrate force field). For frag3, the aminopentyl spacer is attached directly to the βdGal side chain branch point, whereas for frag4 and frag5 it is attached to βlRha, with the αdGal side chain likely acting as a buffer as well as protecting against conformational changes of the βdGal (1→4)βlRha glycosidic linkage. Ass discussed above, this linkage is much more flexible in Ss1 and Ss14, which lack the αdGal side chain.

Interestingly, in all cases the side chain fragments are less flexible than the side chain in the Ss2 CPS, with the antigens therefore more conformationally defined in the fragments: compare the Ss2 C1 conformation at 26%, with 70, 86 and 66% for C1 in frag3, frag4 and frag5, respectively. This significant difference indicates that the carbohydrate backbone drives some conformational changes in the CPS side chains.

## Discussion

This modeling study of four related *S. suis* CPS revealed that the backbone conformation and dynamics differs markedly in Ss2 and Ss1/2 relative to Ss1 and Ss14. Because a well-defined helical conformation (Figures 3A,B), with side chains arranged on the outer edge, shields the backbone residues from solvent exposure, we find that the CPS backbone is an unlikely source of antibody epitopes for Ss2 and Ss1/2. This provides an explanation for the backbone of Ss1/2 and Ss2 surprisingly not being involved in the cross-reaction between these two serotypes (Van Calsteren et al., 2016). On the other hand, Ss1 and Ss14 have a highly flexible backbone, which also lacks the shielding αdGal side chain, and thus is relatively exposed for antibody binding. For this reason, the backbone is potentially a common epitope for antibody binding to the Ss1 and Ss14 CPS, as previously suggested (Van Calsteren et al., 2016).

Further, all the changes in the Ss1/Ss14 backbone relative to the Ss2 and Ss1/2 backbone seem designed to enhance the flexibility of the CPS chain. We can therefore speculate, as we did for our modelling study of Group B *Streptococcus* type III (GBSIII), that this is a strategy for bacterial evasion of the host immune system in these serotypes: a flexible backbone with multiple conformations that presents a “moving target” to the immune system, combined with conformationally-defined human-mimic sialic-acid containing epitopes (Kuttel and Ravenscroft 2019). However, while the expression of sialic acid is essential for elicitation of protective antibodies against GBSIII (Carboni et al., 2020), it is not essential for the four *S. suis* CPS explored here. Modelling provides a mechanistic rationale for this surprising result: in GBSIII the αdNeu5Ac(2→3) linkage results in exposure of this epitope on the end of the short side chain, whereas the more flexible αdNeu5Ac(2→6) linkage in the *S. suis* side chain is preferentially in a hairpin bend conformation. This hairpin bend exposes a sialic-acid epitope (termed EpA, Figure 4A) that is common across all four CPS and thus provides a mechanistic rationale for the previously identified monoclonal antibody (mAb 9E7) of relatively high avidity that interacts with the side chain of all four CPS (Goyette-Desjardins et al., 2019). However, an alternate side chain epitope provides the possibility for antibodies to avoid sialic acid binding in *S. suis.* We identified a second epitope on the other side of the hairpin bend, which we term EpB, that is independent of the sialic acid. Our modelling showed that this epitope is preserved in the three Ss2 vaccine candidate oligosaccharide fragments, even when the sialic acid is absent. Antibody binding to the EpB epitope therefore provides a mechanistic rationale for the efficacy of the frag4 pentasaccharide that does not contain sialic acid, but has still been shown to be a promising vaccine candidate against Ss2 (Zhang et al., 2021). Such an epitope that excludes sialic acid may be selected for in the mammalian immune response, where sialic acid has immunomodulatory properties (Calzas et al., 2017).

However, unlike the EpA epitope, the EpB epitope is not maintained across the four CPS. EpB is significantly altered by the substitution of the βdGal residue in Ss2 and Ss14 for a βdGalNAc in Ss1/2 and Ss1: the N-acetyl substitution is highly exposed on the hairpin bend, forming the EpB* epitope. Asymmetrical cross-reactions previously identified between *S. suis* sera (Van Calsteren et al., 2016) may be explained in terms of one serum recognizing the common EpA epitope, whereas the other serum recognizes the EpB* epitope. Further, serological cross-reactivity between Ss1/2 and Ss1, but not between anti-Ss1/2 serum and Ss2, as well as limited cross-reactivity between anti-Ss1 serum and Ss14, provides a strong indication that the EpB* epitope is immunodominant (Van Calsteren et al., 2016). Furthermore, we identified a secondary extended side chain conformation exposing an epitope comprising the terminal sialic acid as well as the adjacent βdGlc (EpC) that is also altered by N-acetylation (to EpC*). Binding of the EpC epitope by the high avidity 13C8 mAb provides a rationalization for its specificity for Ss2 and Ss14, which is removed by desialylation (Goyette-Desjardins et al., 2019). It is likely that the Ss2 specific mAb 16h11 identified also binds the EpC epitope, but a larger fragment including the αlRha.

Encouragingly, we found that the three vaccine candidate oligosaccharide fragments have dominant conformations that are remarkably consistent with the Ss2 CPS sidechain. However, investigation of the effect of the aminopentyl spacer on the conformation of small synthetic oligosaccharides is warranted, as this work suggests that the spacer affects the conformation of the tetrasaccharide frag3, with the αdGal side chain in frag4 and frag5 acting as a buffer between the spacer and the functional part of the epitopes, as well as protecting against conformational changes of the βdGal(1→4)βlRha glycosidic linkage. Modelling of this spacer with the vaccine candidates is possible, but will require adjustments to the forcefield to support this substitution.

Finally, the varying flexibilities found for the molecules modelled in this study may have relevance for their observed immunogenicity: our prior modelling study of meningococcal serogroups Y and W (Kuttel et al., 2017) suggested a relationship between reduced carbohydrate chain flexibility (serotype Y) and improved cross protection with closely related, more flexible, serotypes (serotype W). We found here that the vaccine candidate oligosaccharides are significantly more conformationally defined than the Ss2 CPS side chain, which may improve their efficacy as vaccine candidates. Further, we found that Ss14 is the most flexible molecule of the four CPS, which may have an adverse impact on its immunogenicity and thus explain why the mAb Z3 cross-reacts with Ss2, Ss1/2 and Ss1, but not with Ss14: the more flexible side chain in Ss14 may not be as effective an epitope in Ss14 as for the other CPS. Extrapolating from this, we propose the αdNeu5Ac(2→6)βdGal**NAc**(1→4)βdGlcNAc(1→3)βdGal(1→**4)[α** **d** **Gal**(**1→3)]β** **l** **Rha** hexasaccharide from Ss1/2 as a vaccine candidate to provide optimal cross-protection against the four closely related *S. suis* serotypes 1, 1/2, 2 and 14, as this fragment is the most conformationally defined and exposes the common EpA epitope as well as the immunodominant EpB* epitope.

## Data Availability

The raw data supporting the conclusions of this article will be made available by the authors, without undue reservation.

## References

[B1] Anish C. Schumann B. Pereira C. L. Seeberger P. H. (2014). Chemical Biology Approaches to Designing Defined Carbohydrate Vaccines. Chem. Biol. 21 (1), 38–50. 10.1016/j.chembiol.2014.01.002 24439205

[B2] Astronomo R. D. Burton D. R. (2010). Carbohydrate Vaccines: Developing Sweet Solutions to Sticky Situations? Nat. Rev. Drug Discov. 9 (4), 308–324. 10.1038/nrd3012 20357803 PMC3878310

[B3] Auger J.-P. Dolbec D. Roy D. Segura M. Gottschalk M. (2018). Role of the Streptococcus Suis Serotype 2 Capsular Polysaccharide in the Interactions with Dendritic Cells Is Strain-dependent but Remains Critical for Virulence. PLoS ONE 13 (7), e0200453. 10.1371/journal.pone.0200453 30001363 PMC6042740

[B4] Calzas C. Taillardet M. Fourati I. S. Roy D. Gottschalk M. Soudeyns H. (2017). Evaluation of the Immunomodulatory Properties of Streptococcus Suis and Group B Streptococcus Capsular Polysaccharides on the Humoral Response. Pathogens 6 (2), 16. 10.3390/pathogens6020016 PMC5488650 28425925

[B5] Carboni F. Angiolini F. Fabbrini M. Brogioni B. Corrado A. Berti F. (2020). Evaluation of Immune Responses to Group B *Streptococcus* Type III Oligosaccharides Containing a Minimal Protective Epitope. J. Infect. Dis. 221 (6), 943–947. 10.1093/infdis/jiz551 31641758

[B6] Cross S. Kuttel M. M. Stone J. E. Gain J. E. (2009). Visualisation of Cyclic and Multi-Branched Molecules with VMD. J. Mol. Graphics Model. 28 (2), 131–139. 10.1016/j.jmgm.2009.04.010 PMC3158682 19473861

[B7] Dutkiewicz J. Zając V. Sroka J. Wasiński B. Cisak E. Sawczyn A. (2018). Streptococcus Suis: a Re-emerging Pathogen Associated with Occupational Exposure to Pigs or Pork Products. Part II - Pathogenesis. Ann. Agric. Environ. Med. 25 (1), 186–203. 10.26444/aaem/85651 29575852

[B8] Dutkiewicz J. Sroka J. Zając V. Wasiński B. Cisak E. Sawczyn A. (2017). Streptococcus Suis: a Re-emerging Pathogen Associated with Occupational Exposure to Pigs or Pork Products. Part I - Epidemiology. Ann. Agric. Environ. Med. 24 (4), 683–695. 10.26444/aaem/79813 29284248

[B9] Feller S. E. Zhang Y. Pastor R. W. Brooks B. R. (1995). Constant Pressure Molecular Dynamics Simulation: the Langevin Piston Method. J. Chem. Phys. 103 (11), 4613–4621. 10.1063/1.470648

[B10] Gottschalk M. (2011). “Streptococcosis,” in Diseases of Swine. Editors Karriker L. Ramirez A. Schwartz K. J. Stevenson G. Zimmerman J. (NJ: Wiley Publishers).

[B11] Gottschalk M. Xu J. Calzas C. Segura M. (2010). Streptococcus Suis: a New Emerging or an Old Neglected Zoonotic Pathogen? Future Microbiol. 5 (3), 371–391. 10.2217/fmb.10.2 20210549

[B12] Goyette-Desjardins G. Auger J. P. Dolbec D. Vinogradov E. Okura M. Takamatsu D. (2020). Comparative Study of Immunogenic Properties of Purified Capsular Polysaccharides from Streptococcus Suis Serotypes 3, 7, 8, and 9: the Serotype 3 Polysaccharide Induces an Opsonizing IgG Response. Infect. Immun. 88 (10), e00377–00320. 10.1128/IAI.00377-20 32747605 PMC7504959

[B13] Goyette-Desjardins G. Auger J.-P. Xu J. Segura M. Gottschalk M. (2014). Streptococcus Suis, an Important Pig Pathogen and Emerging Zoonotic Agent-An Update on the Worldwide Distribution Based on Serotyping and Sequence Typing. Emerging Microbes & Infections 3 (1), 1–20. 10.1038/emi.2014.45 PMC4078792 26038745

[B14] Goyette-Desjardins G. Calzas C. Shiao T. C. Neubauer A. Kempker J. Roy R. (2016). Protection against Streptococcus Suis Serotype 2 Infection Using a Capsular Polysaccharide Glycoconjugate Vaccine. Infect. Immun. 84 (7), 2059–2075. 10.1128/iai.00139-16 27113360 PMC4936365

[B15] Goyette-Desjardins G. Lacouture S. Auger J.-P. Roy R. Gottschalk M. Segura M. (2019). Characterization and Protective Activity of Monoclonal Antibodies Directed against Streptococcus Suis Serotype 2 Capsular Polysaccharide Obtained Using a Glycoconjugate. Pathogens 8 (3), 139. 10.3390/pathogens8030139 PMC6789524 31500262

[B16] Grossfield A. Zuckerman D. M. (2009). Chapter 2 Quantifying Uncertainty and Sampling Quality in Biomolecular Simulations. Annu. Rep. Comput. Chem. 5, 23–48. 10.1016/s1574-1400(09)00502-7 20454547 PMC2865156

[B17] Guvench O. Mallajosyula S. S. Raman E. P. Hatcher E. Vanommeslaeghe K. Foster T. J. (2011). CHARMM Additive All-Atom Force Field for Carbohydrate Derivatives and its Utility in Polysaccharide and Carbohydrate-Protein Modeling. J. Chem. Theor. Comput. 7 (10), 3162–3180. 10.1021/ct200328p PMC3224046 22125473

[B18] Hoover W. G. (1985). Canonical Dynamics: Equilibrium Phase-Space Distributions. Phys. Rev. A. 31 (3), 1695–1697. 10.1103/physreva.31.1695 9895674

[B19] Humphrey W. Dalke A. Schulten K. (1996). VMD: Visual Molecular Dynamics. J. Mol. Graphics 14 (1), 33–38. 10.1016/0263-7855(96)00018-5 8744570

[B20] Hunter J. D. (2007). Matplotlib: A 2D Graphics Environment. Comput. Sci. Eng. 9 (3), 90–95. 10.1109/mcse.2007.55

[B21] Jorgensen W. L. Chandrasekhar J. Madura J. D. Impey R. W. Klein M. L. (1983). Comparison of Simple Potential Functions for Simulating Liquid Water. J. Chem. Phys. 79 (2), 926–935. 10.1063/1.445869

[B22] Kuttel M. M. Casadevall A. Oscarson S. (2020). *Cryptococcus Neoformans* Capsular GXM Conformation and Epitope Presentation: A Molecular Modelling Study. Molecules 25 (11), 2651. 10.3390/molecules25112651 PMC7321252 32517333

[B23] Kuttel M. M. Ravenscroft N. (2018). “The Role of Molecular Modeling in Predicting Carbohydrate Antigen Conformation and Understanding Vaccine Immunogenicity” in Carbohydrate-Based Vaccines: From Concept to Clinic. Washington, DC: ACS Publications, 139–173. 10.1021/bk-2018-1290.ch007

[B24] Kuttel M. M. Ståhle J. Widmalm G. (2016). CarbBuilder: Software for Building Molecular Models of Complex Oligo- and Polysaccharide Structures. J. Comput. Chem. 37 (22), 2098–2105. 10.1002/jcc.24428 27317625

[B25] Kuttel M. M. Timol Z. Ravenscroft N. (2017). Cross-protection in Neisseria Meningitidis Serogroups Y and W Polysaccharides: A Comparative Conformational Analysis. Carbohydr. Res. 446-447, 40–47. 10.1016/j.carres.2017.05.004 28501716

[B26] Kuttel M. Ravenscroft N. (2019). Conformation and Cross-Protection in Group B Streptococcus Serotype III and Streptococcus Pneumoniae Serotype 14: A Molecular Modeling Study. Pharmaceuticals 12 (1), 28. 10.3390/ph12010028 PMC6469160 30781826

[B27] Lecours M.-P. Fittipaldi N. Takamatsu D. Okura M. Segura M. Goyette-Desjardins G. (2012). Sialylation of Streptococcus Suis Serotype 2 Is Essential for Capsule Expression but Is Not Responsible for the Main Capsular Epitope. Microbes Infect. 14 (11), 941–950. 10.1016/j.micinf.2012.03.008 22521569

[B28] Lockhart S. P. Scott D. A. Jansen K. U. Anderson A. S. Gruber W. C. (2018). “Glycoconjugate Vaccines: The Clinical Journey” in Carbohydrate-Based Vaccines: From Concept to Clinic. Washington, DC: ACS Publications, 7–59. 10.1021/bk-2018-1290.ch002

[B29] Nosé S. Klein M. (1983). Constant Pressure Molecular Dynamics for Molecular Systems. Mol. Phys. 50 (5), 1055–1076.

[B30] Phillips J. C. Braun R. Wang W. Gumbart J. Tajkhorshid E. Villa E. (2005). Scalable Molecular Dynamics with NAMD. J. Comput. Chem. 26 (16), 1781–1802. 10.1002/jcc.20289 16222654 PMC2486339

[B31] Richardson N. I. Kuttel M. M. Michael F. S. Cairns C. Cox A. D. Ravenscroft N. (2021). Cross-reactivity of *Haemophilus Influenzae* Type a and B Polysaccharides: Molecular Modeling and Conjugate Immunogenicity Studies. Glycoconj. J., 1–12. 10.1007/s10719-021-10020-0 34491462

[B32] Segura M. Calzas C. Grenier D. Gottschalk M. (2016). Initial Steps of the Pathogenesis of the Infection Caused byStreptococcus Suis: Fighting against Nonspecific Defenses. FEBS Lett. 590 (21), 3772–3799. 10.1002/1873-3468.12364 27539145

[B33] Segura M. (2015). Streptococcus Suisvaccines: Candidate Antigens and Progress. Expert Rev. Vaccin. 14 (12), 1587–1608. 10.1586/14760584.2015.1101349 26468755

[B34] Stone J. E. Phillips J. C. Freddolino P. L. Hardy D. J. Trabuco L. G. Schulten K. (2007). Accelerating Molecular Modeling Applications with Graphics Processors. J. Comput. Chem. 28 (16), 2618–2640. 10.1002/jcc.20829 17894371

[B35] Van Calsteren M.-R. Gagnon F. Calzas C. Goyette-Desjardins G. Okura M. Takamatsu D. (2013). Structure Determination ofStreptococcus Suisserotype 14 Capsular Polysaccharide. Biochem. Cel Biol. 91 (2), 49–58. 10.1139/bcb-2012-0036 23527632

[B36] Van Calsteren M.-R. Gagnon F. Lacouture S. Fittipaldi N. Gottschalk M. (2010). Structure Determination ofStreptococcus Suisserotype 2 Capsular Polysaccharide. Biochem. Cel Biol. 88 (3), 513–525. 10.1139/o09-170 20555393

[B37] Van Calsteren M.-R. Goyette-Desjardins G. Gagnon F. Okura M. Takamatsu D. Roy R. (2016). Explaining the Serological Characteristics of Streptococcus Suis Serotypes 1 and 1/2 from Their Capsular Polysaccharide Structure and Biosynthesis. J. Biol. Chem. 291 (16), 8387–8398. 10.1074/jbc.m115.700716 26912653 PMC4861414

[B38] Varki A. Cummings R. D. Aebi M. Packer N. H. Seeberger P. H. Esko J. D. (2015). Symbol Nomenclature for Graphical Representations of Glycans. Glycobiology 25 (12), 1323–1324. 10.1093/glycob/cwv091 26543186 PMC4643639

[B39] Woods R. J. (2018). Predicting the Structures of Glycans, Glycoproteins, and Their Complexes. Chem. Rev. 118 (17), 8005–8024. 10.1021/acs.chemrev.8b00032 30091597 PMC6659753

[B40] Zhang S. Sella M. Sianturi J. Priegue P. Shen D. Seeberger P. H. (2021). Discovery of Oligosaccharide Antigens for Semi‐Synthetic Glycoconjugate Vaccine Leads against Streptococcus Suis Serotypes 2, 3, 9 and 14**. Angew. Chem. Int. Ed. 60 (26), 14679–14692. 10.1002/anie.202103990 PMC8252040 33852172

