# Supplementary material for: Comparative Molecular Modelling of Capsular Polysaccharide Conformations in Streptococcus suis Serotypes 1, 2, 1/2 and 14 Identifies Common Epitopes for Antibody Binding
Source: Front Mol Biosci. 2022 Feb 8;9:830854. doi: 10.3389/fmolb.2022.830854 (PMC8861514; doi:10.3389/fmolb.2022.830854)
Supplement: Supplementary file 1 [file DataSheet1.docx]

Supplementary Material

# Supplementary Figures


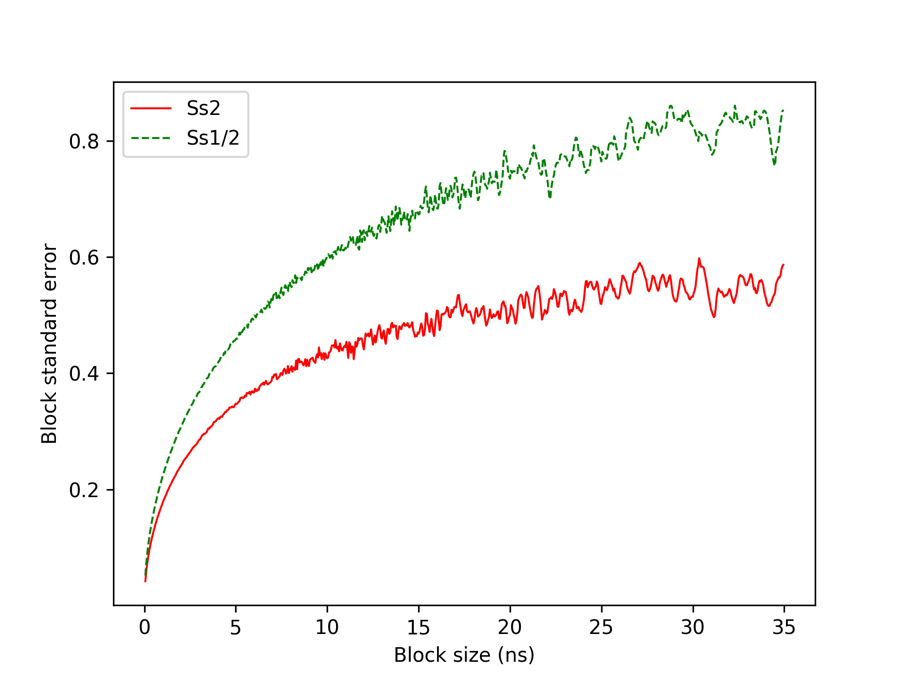


**Supplementary Figure S1.** Block standard error (BSE) analysis of the time series of the end-to-end distance for the 1 μs simulations of the 6RU Ss2 and Ss1/2 CPS, indicating simulation convergence. The BSE estimated correlation times are 9 ns (Ss2) and 12 ns (Ss1/2), and the numbers of independent samples times are 109 (Ss2) and 81 (Ss1/2).


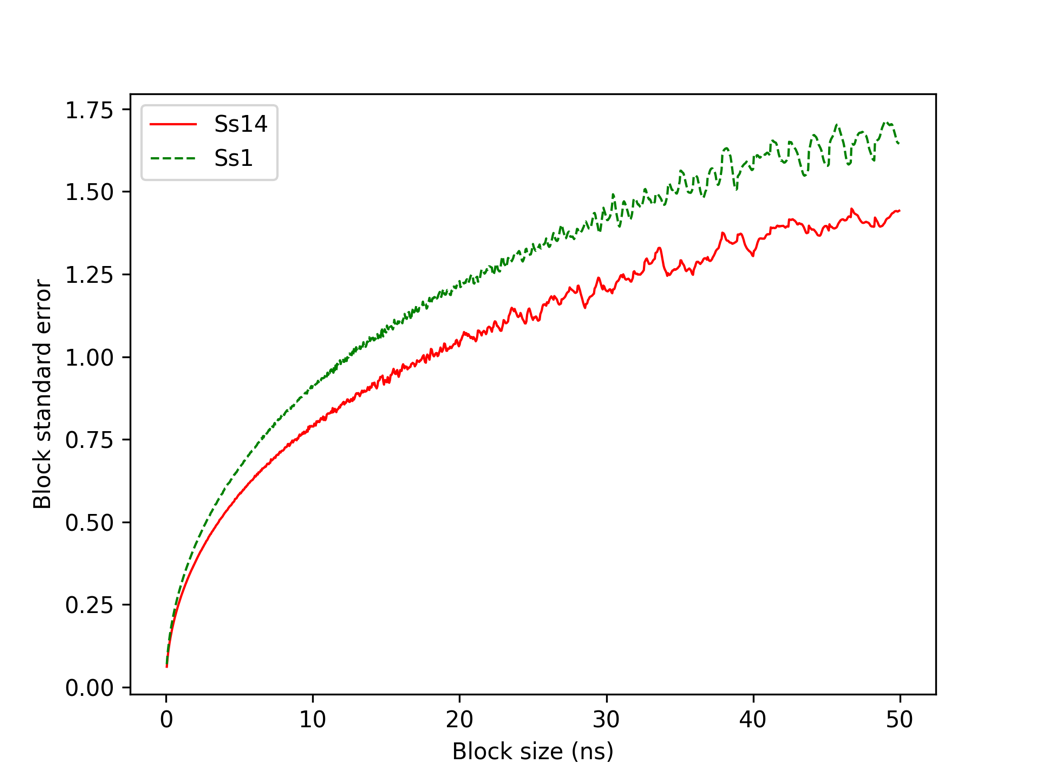


**Supplementary Figure S2.** Block standard error (BSE) analysis of the time series of the end-to-end distance for the 1.4 μs simulations of the 6RU Ss14 and Ss1 CPS, indicating simulation convergence. The BSE estimated correlation times are 27 ns (Ss14) and 29 ns (Ss1), and the numbers of independent samples times are 51 (Ss14) and 49 (Ss1).


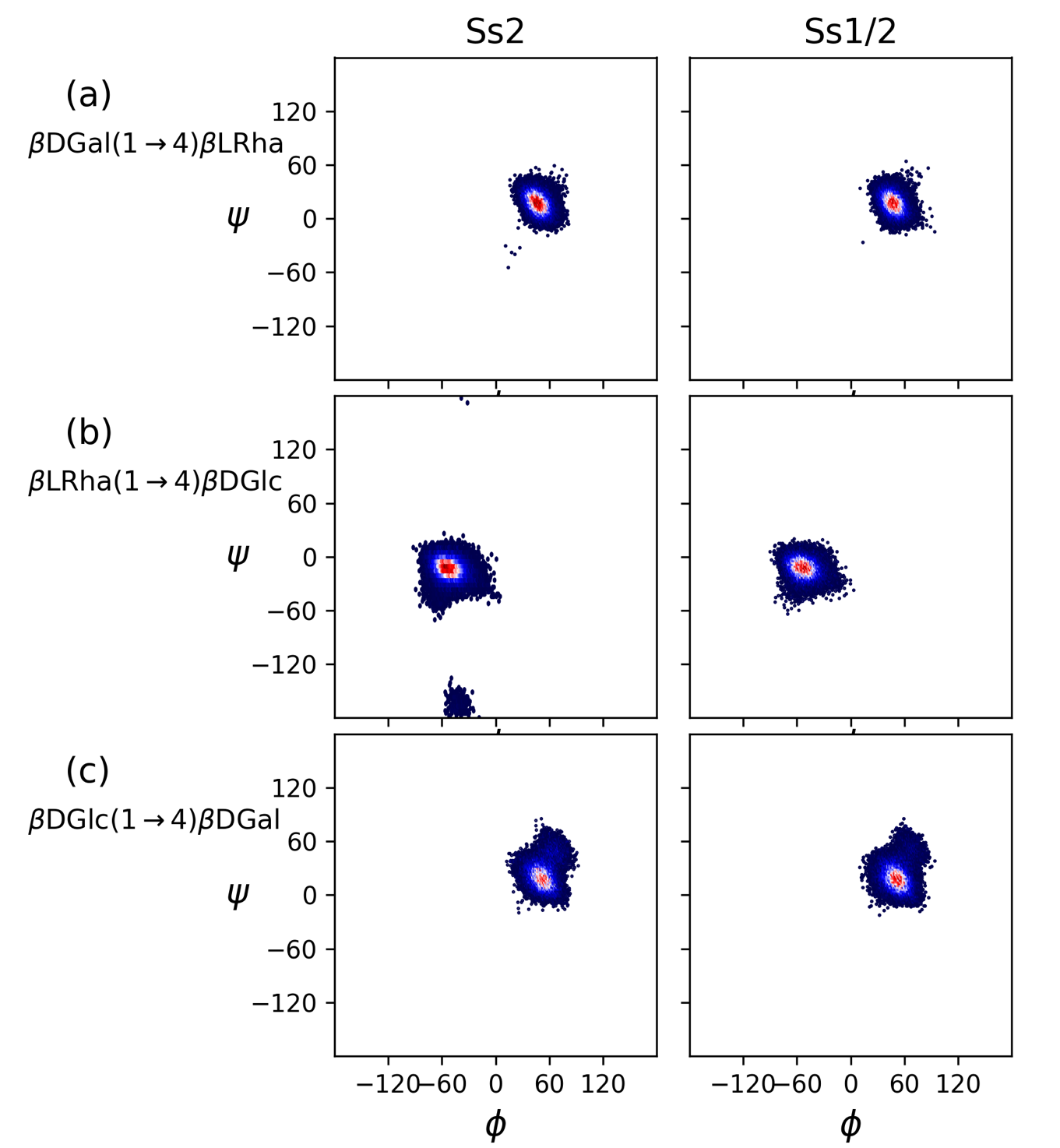


**Supplementary Figure S3.** Heat maps of the backbone glycosidic linkage orientation distribution in the two central RUs (RU3 and RU4) for the simulations of 6RU CPS chains of Ss2 and Ss1/2. Row (a) shows the backbone βdGal(1→4)βlRha linkage, row (b) the βlRha(1→4)βdGlc linkage and row (c) the βdGlc(1→4) βdGal linkage. Glycosidic linkage orientations are shown as rotations of the linkage dihedral angles φ and ψ.


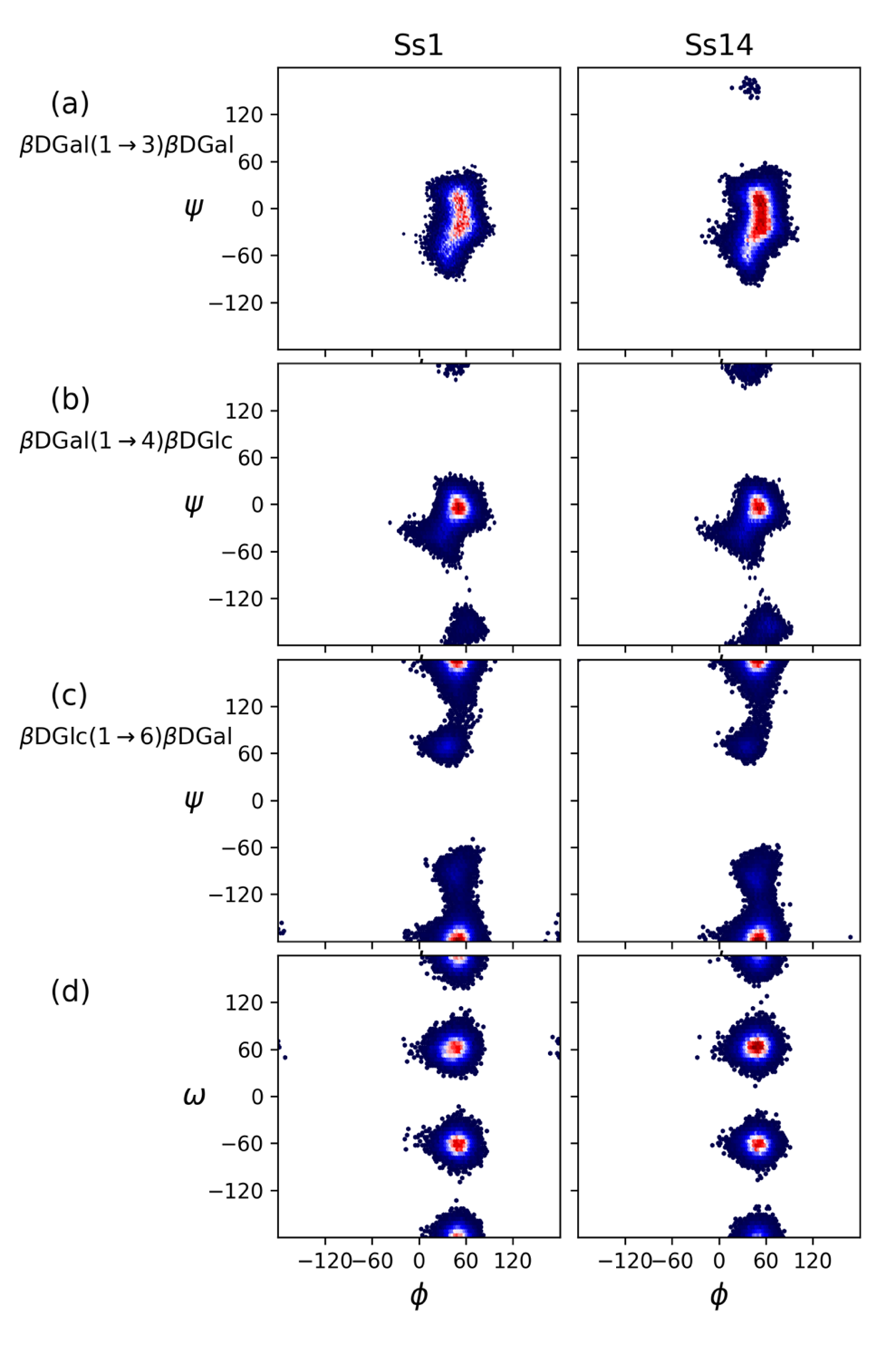


**Supplementary Figure S4.** Heat maps of the backbone glycosidic linkage orientation distribution in the two central RUs (RU3 and RU4) for the simulations of 6RU CPS chains of Ss1 and Ss14. Row (a) shows the backbone βdGal(1→3)βdGal linkage, row (b) the βdGal(1→4)βdGlc linkage and the last two rows the three bond βdGlc(1→6) βdGal linkage. Glycosidic linkage orientations are shown as rotations of the linkage dihedral angles φ and ψ, except for the last row which is φ and ψ.

**
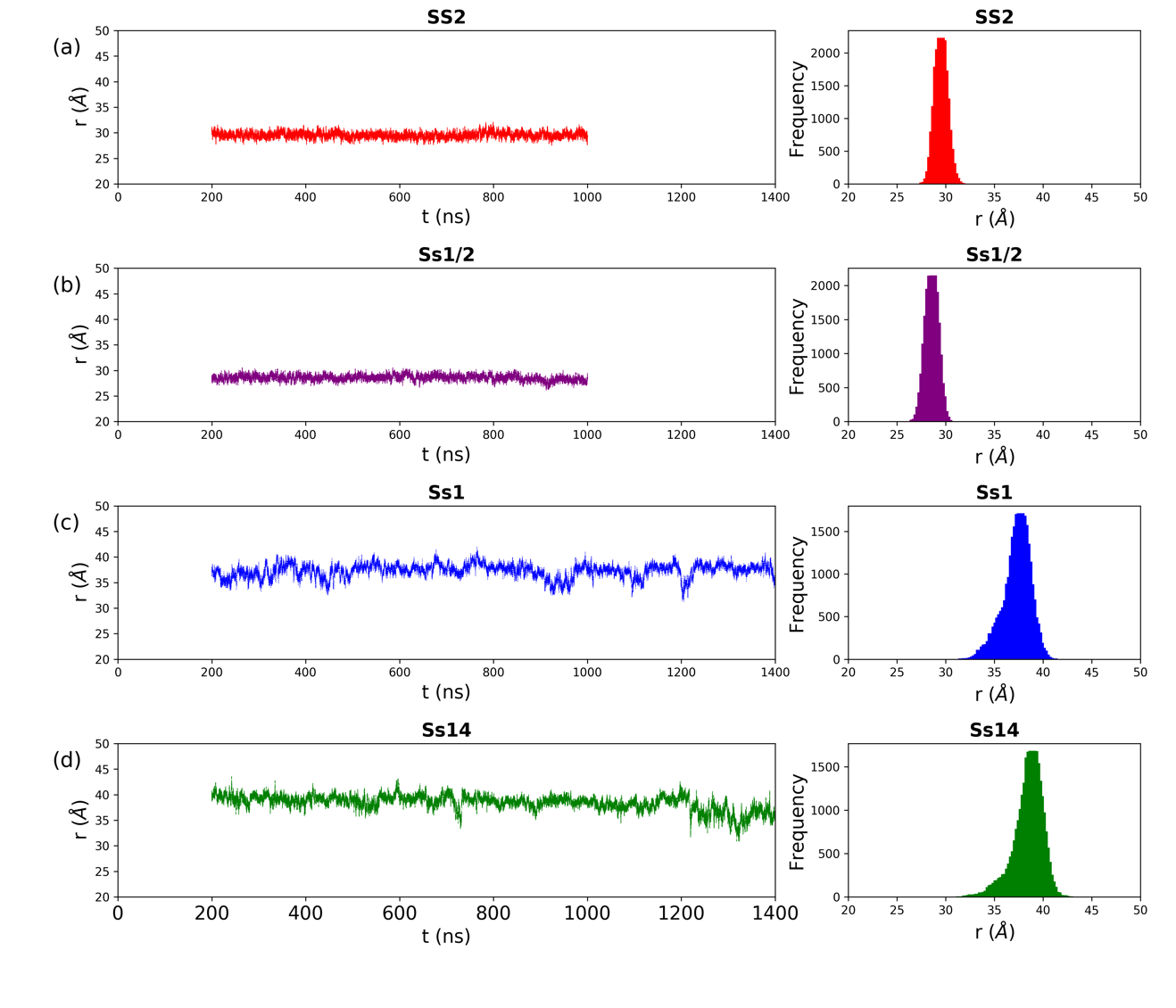
**

**Supplementary Figure S5.** Time series and histogram plots of the percentage molecular solvent accessible surface area for the backbone residues relative to the entire CPS for : (a) Ss2, (b) Ss1/2, (c) Ss1 and (d) Ss14. Note that for the more flexible Ss1 and Ss14, longer simulation times were required to reach convergence (both 1400 ns) than for Ss2 and Ss1/2 (both 1000 ns).


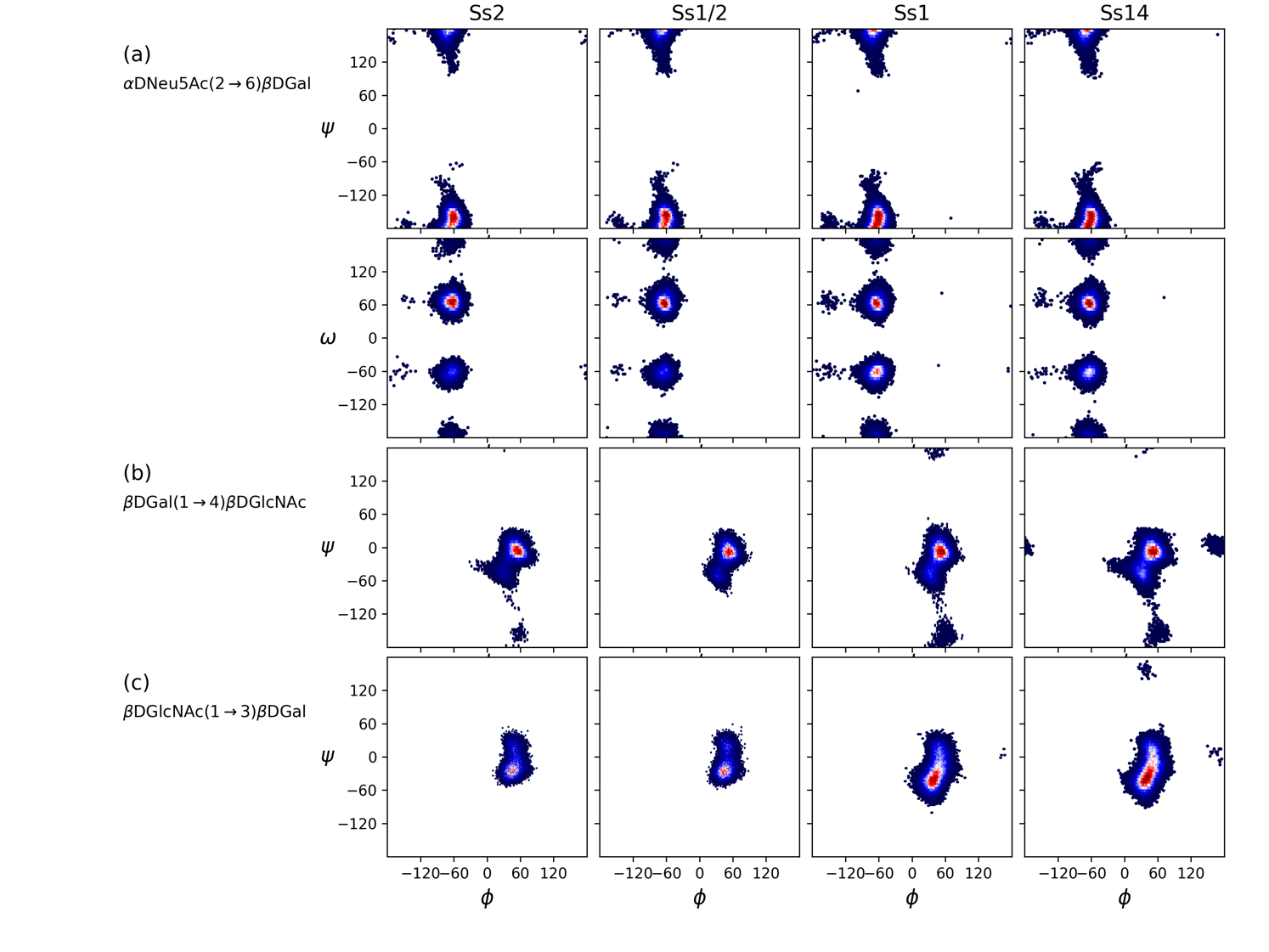


**Supplementary Figure S6.** Heat maps of the side chain glycosidic linkage orientation distribution in the two central RUs (RU3 and RU4) for the simulations of 6RU CPS chains of Ss2 (column 1), Ss1/2 (column 2), Ss1 (column 3) and Ss14 (column 4). The two rows in (a) show the maps for the three-bond αdNeu5Ac(2→6)βdGal**NAc** linkage, row (b) the βdGal**NAc**(1→4)βdGlcNAc linkage and row (c) the βdGlcNAc(1→3)βdGal linkage. Glycosidic linkage orientations are shown as rotations of the linkage dihedral angles φ and ψ, except for the second row, which is φ and ψ.


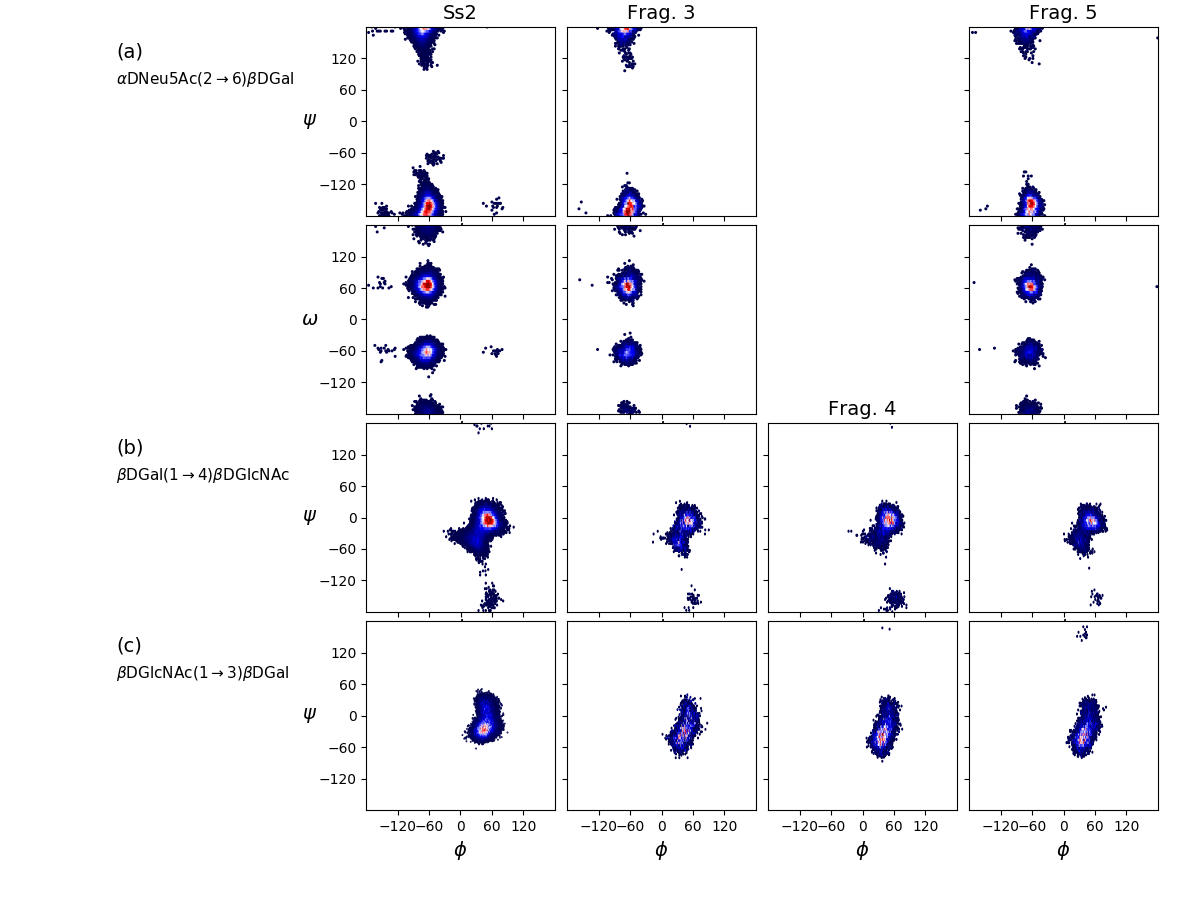


**Supplementary Figure S6.** Heat maps of the side chain glycosidic linkage orientation distribution for the simulations of the 6RU Ss2 CPS (column 1, dihedrals from RU3 and RU4) compared with the three synthentic vaccine candidates from Zhang et al.: the tetrasaccharide frag3 (column 2), the pentasaccharide frag4 (column 3) and the hexasaccharide frag5 (column 4). The two rows in (a) show the maps for the three-bond αdNeu5Ac(2→6)βdGal**NAc** linkage (absent in frag4), row (b) the βdGal**NAc**(1→4)βdGlcNAc linkage and row (c) the βdGlcNAc(1→3)βdGal linkage. Glycosidic linkage orientations are shown as rotations of the linkage dihedral angles φ and ψ, except for the second row, which is φ and ψ.
